# ﻿Addition of three new species of Xylariomycetidae fungi on bamboo from Southern China

**DOI:** 10.3897/mycokeys.109.128020

**Published:** 2024-10-02

**Authors:** Xin Zhou, Kamran Habib, Wenyu Zeng, Yulin Ren, Xiangchun Shen, Jichuan Kang, Qirui Li

**Affiliations:** 1 State Key Laboratory of Functions and Applications of Medicinal Plants, Guizhou Medical University, Gui'an, Guizhou, 561113, China Guizhou Medical University Gui'an China; 2 The High Efficacy Application of Natural Medicinal Resources Engineering Centre of Guizhou Province (The Key Laboratory of Optimal Utilization of Natural Medicine Resources), School of Pharmaceutical Sciences, Guizhou Medical University, Gui’an, Guizhou, 561113, China Khushal Khan Khattak University Karak Pakistan; 3 Department of Botany, Khushal Khan Khattak University, Karak, KP, 27200 Pakistan Guizhou Medical University Gui’an China; 4 Engineering and Research Centre for Southwest Bio-Pharmaceutical, Resources of National Education Ministry of China, Guizhou University, Guiyang, Guizhou, 550025, China Guizhou University Guiyang China

**Keywords:** Cainiaceae, Guizhou, systematics, Xylariales

## Abstract

In our ongoing research on bambusicolous Xylariomycetidae fungi, three new microfungi taxa were collected and identified as members of the genera *Amphibambusa*, *Arecophila*, and *Nigropunctata*. *Amphibambusaaureae***sp. nov.**, *Arecophilagaofengensis***sp. nov.**, and *Nigropunctataxiaohensis***sp. nov.** are introduced based on morphological comparisons and phylogenetic analyses using combined ITS, LSU, *tub2*, and *tef*1α loci. Comprehensive morphological descriptions, illustrations, and a phylogenetic tree showcasing the placement of these new taxa are provided. Additionally, keys to *Amphibambusa* and *Nigropunctata* are provided.

## ﻿Introduction

Bamboo, as the largest member of the grass family Poaceae, plays an important role in local economies worldwide, being distributed across diverse climates, from cold mountainous regions to hot tropical areas. Bamboos exhibit high diversity and are particularly abundant in Asia, notably in China. China boasts plentiful bamboo resources, with its bamboo species constituting of more than 50% of the world’s total (Liu et al. 2018). Due to their low natural toxicity, bamboos are susceptible to fungi and insect infestations resulting in abundant microfungi inhabiting their culms and leaves (Dai et al. 2018; Wang et al. 2018; Jiang et al. 2022; Hyde et al. 2023a, b).

Dai et al. (2018) reported an association of more than 1300 fungi with bamboo, consisting of 150 basidiomycetes and 800 ascomycetes species. Among these, 240 and 110 taxa have been reported as hyphomycetous and coelomycetous, respectively. The taxonomic placements of bamboo-associated ascomycetous fungi are highly diverse, comprising more than 1150 species, in 120 families and 400 genera (Dai et al. 2018). Among these families, Xylariaceae and Hypocreaceae are the most abundant, with 74 (belonging to 18 genera) and 63 (belonging to 14 genera) species, respectively (Dai et al. 2018; Wijayawardene et al. 2022). The genus *Phyllachora* holds the highest number of species occurring on bamboo, followed by *Nectria* and *Hypoxylon* (Dai et al. 2018). Most bambusicolous ascomycetous taxa in China are known from Taiwan, with 144 species, followed by Hong Kong with 139 species, Yunnan with 133 species, Guangdong with 53 species, Zhejiang with 37 species, Jiangsu with 36 species, and Sichuan with 35 species (Jiang et al. 2022). Jiang et al. (2022) reported 512 bambusicolous ascomycetous taxa from China, associated with 16 bamboo genera. These species are distributed across 50 orders, 116 families, and 279 genera (including 45 genera without any higher rank) and represent more than one-third of the known bambusicolous ascomycetes in the world. Most reported bambusicolous fungi lack detailed morphology or sequence data thus, still require further study (Dai et al. 2018).

During the investigation of bambusicolous Xylariomycetidae fungi, we observed specimens that could not be readily assigned to any known species. To better understand their taxonomic position, we conducted a phylogenetic analysis using a multi-marker approach (internal transcribed spacer ITS, large subunit LSU, β-tubulin *tub2*, and translation elongation factor *tef*1α). Their distinct morphological characteristics distinguish them from the known species. As a result, we propose these specimens as new species.

## ﻿Materials and methods

### ﻿Sample collection and morphological study

The specimens were collected during surveys conducted in Guizhou province, and Guangxi Zhuang Autonomous Region in China during 2023. All related habitat information was recorded. The photos of the collected materials were taken using a Canon G15 camera (Canon Corporation, Tokyo, Japan). Materials were placed in paper bags and taken to the lab for morphological characterization and isolation. To preserve the freshness of the specimens, they were dried using a portable fan drier. The dried specimens were carefully labeled and stored. After this preparation, the specimens were ready for both morphological and molecular studies. All specimens were deposited at the Herbarium of Guizhou Medical University (**GMB**) and the Herbarium of Cryptogams, Herbarium of Kunming Institute of Botany, Chinese Academy of Sciences (**KUN-HKAS**), living cultures were deposited at the Guizhou Medical University Culture Collection (**GMBC**).

### ﻿Morphological characterization and isolation

Macroscopic features (ostiole, clypeus, etc.) of the specimens were examined using an Olympus SZ61 stereomicroscope and photographed using a Canon 700D digital camera. Microscopic morphological features (ascomata, peridium, paraphyses, asci, ascospore, etc.) were observed using an optical microscope (Nikon Ni) and photographed using a Canon 700D digital camera attached. Melzer’s iodine reagent was used to test the apical apparatus structures for amyloid reaction. Asci and ascospores of the samples were measured using Tarosoft Image Framework (v. 0.9.0.7). Images were polished using Adobe Photoshop CS6 (Adobe Systems, USA). Pure cultures were obtained by single-ascospore isolation (Long et al. 2019) and maintained at 25 °C for 1–5 weeks on PDA (potato dextrose agar) and oatmeal-agar (OA) medium.

### ﻿DNA extraction, PCR amplification and sequencing

Mycelium was scraped from pure culture plates using a sterilized scalpel and was used for DNA extraction with the methods following the manufacturer’s instructions of the BIOMIGA fungus genomic DNA extraction kit. For some specimens where the ascospores did not germinate, we used a method of directly extracting DNA from the contents of the perithecium. The DNA samples were kept at –20 °C. Internal transcribed spacers (ITS), large subunit LSU, β-tubulin (*tub2*), and translation elongation factor (*tef*1α) were amplified by PCR with primers ITS1/ITS4 (White et al. 1990; Gardes and Bruns 1993), LR0R/LR5 (Vilgalys and Hester 1990), Bt2a/Bt2b (Glass and Donaldson 1995), and EF1-983F/EF1-2218R (Rehner and Buckley 2005), respectively. The components of a 25 μL volume PCR mixture was: 9.5 μL of double distilled water, 12.5 μL of PCR Master Mix, 1 μL of each primer, and 1 μL of template DNA. Qualified PCR products were checked through 1.5% agarose gel electrophoresis stained with Golden View, and were sent to Sangon Co., China, for sequencing (Xie et al. 2020).

### ﻿Sequence alignments and phylogenetic analyses

All the obtained sequences were deposited in the GenBank (Tables 1, 2). These sequences were compared with each other and all the known sequences in GenBank using the BLASTN algorithm for precise identification. The molecular phylogeny was inferred from a combined dataset of ITS, LSU, *tub2* and *tef*1α sequences. The reference sequences retrieved from open databases originated from recent published literature, and the Blastn results of close matches. Sequences were aligned using the MAFFT v.7.110 online program (Katoh et al. 2019) with the default settings, respectively. The alignment was adjusted manually using BioEdit v.7.0.5.3 (Hall 1999) where necessary. The maximum likelihood (ML) analysis was implemented in RAxML v.8.2.12 using the GTRGAMMA substitution model with 1,000 bootstrap replicates (Stamatakis 2014). The phylogenetic analyses were also performed for Bayesian inference in MrBayes v. 3.2.1 (Ronquist et al. 2012) online. The Markov Chain Monte Carlo (MCMC) sampling in MrBayes v.3.2.2 (Ronquist et al. 2012) was used to determine the posterior probabilities (PP). Six simultaneous Markov chains were run for 1,000,000 generations, and trees were sampled every 1,000^th^ generation. The phylogenetic tree was visualized in FIGTREE v.1.4.4 (Rambaut 2018). All analyses were run on the CIPRES Science Gateway v 3.3 webportal (Miller et al. 2010).

**Table 1.** Taxa and corresponding GenBank accession numbers of sequences used in the phylogenetic analysis of Fig. 1.

Species	Strain number	GenBank Accession Numbers		References
		ITS	LSU	
***Amphibambusaaureae***	**GMB4550^T^**	**PQ066508**	**PQ066514**	**The study**
***Amphibambusaaureae***	**GMB4561**	**PQ066509**	**PQ066515**	**The study**
*Amphibambusabambusicola*	MFLLUCC 11-0617^T^	KP744433	KP744474	Liu et al. (2015)
*Amphibambusahongheensis*	KUN-HKAS 112723^T^	MW892971	MW892969	Jiang et al. (2021a)
*Amphibambusahongheensis*	KUNMCC 20-0334^T^	MW892972	MW892970	Jiang et al. (2021a)
*Arecophilaaustralis*	GZUCC0124	MT742125	MT742132	Li et al. (2022)
*Arecophilaaustralis*	GZUCC0112^T^	MT742126	MT742133	Li et al. (2022)
*Arecophilabambusae*	HKUCC 4794	NA	AF452038	Kang et al. (1999)
*Arecophilaclypeata*	GZUCC0127	MT742128	MT742135	Li et al. (2022)
*Arecophilaclypeata*	GZUCC0110^T^	MT742129	MT742136	Li et al. (2022)
***Arecophilagaofengensis***	**GMB4541^T^**	**PQ066512**	**PQ066516**	**The study**
***Arecophilagaofengensis***	**GMB4559**	**PQ066513**	**PQ066517**	**The study**
*Arecophilamiscanthii*	MFLU 19-2333^T^	NR171235	NG088086	Hyde et al. (2020a)
*Arecophilamiscanthii*	FU31025	MK503821	MK503827	Hyde et al. (2020a)
*Arecophilamuroiana*	GZUCC0122	MT742127	MT742134	Li et al. (2022)
*Arecophilazhaotongensis*	ZHKU 23-0260	OR995738	OR995745	Han et al. (2024)
*Arecophilazhaotongensis*	ZHKU 23-0259	OR995735	OR995742	Han et al. (2024)
*Arecophilazhaotongensis*	GMBCC1145^T^	OR995740	OR995747	Han et al. (2024)
*Arecophila* sp.	HKUCC 6487	NA	AF452039	Jeewon et al. (2003)
*Arecophilaxishuangbannaensis*	ZHKU 23-0280	OR995737	OR995744	Han et al. (2024)
*Arecophilaxishuangbannaensis*	GMB-W1283^T^	OR995736	OR995743	Han et al. (2024)
*Atrotorquatalineata*	Mt25	AF009807	NA	Kang et al. (1998)
*Barrmaeliamacrospora*	CBS 142768^T^	NR167684	NA	Jaklitsch et al. (2014)
*Barrmaeliarhamnicola*	CBS 142772^T^	NR153497	NA	Voglmayr et al. (2018)
*Cainiaanthoxanthis*	MFLUCC 15-0539^T^	NR138407	NG070382	Senanayake et al. (2015)
*Cainiadesmazieri*	CAI	KT949896	NA	Jaklitsch et al. (2016)
*Cainiaglobosa*	MFLUCC 13-0663^T^	NR171724	KX822123	Hyde et al. (2016)
*Cainiagraminis*	CBS 136.62	MH858123	MH869701	Vu et al. (2019)
*Endocalyxcinctus*	JCM 7946	LC228648	LC228704	Delgado et al. (2022)
*Endocalyxcinctus*	NBRC 31306	MZ313191	MZ313152	Delgado et al. (2022)
*Endocalyxgrossus*	JCM 5164^T^	MZ313160	MZ313138	Delgado et al. (2022)
*Endocalyxgrossus*	JCM 5165	MZ313159	MZ313158	Delgado et al. (2022)
*Endocalyxgrossus*	JCM 5166	MZ313179	MZ313171	Delgado et al. (2022)
*Endocalyxindumentum*	JCM 5171^T^	MZ313153	MZ313161	Delgado et al. (2022)
*Endocalyxindumentum*	JCM 8042	MZ313162	MZ313157	Delgado et al. (2022)
*Endocalyxmelanoxanthus*	CBS 147393	MW718204	NA	Delgado et al. (2022)
*Endocalyxmelanoxanthus*	CBS 147394	MW718203	NA	Delgado et al. (2022)
*Endocalyxmetroxyli*	MFLUCC 15-0723B	MT929163	MT929314	Konta et al. (2021)
*Endocalyxmetroxyli*	MFLUCC 15-0723A^T^	NR176745	MT929313	Konta et al. (2021)
*Endocalyxmetroxyli*	MFLUCC 15-0723C	NA	MT929315	Konta et al. (2021)
*Endocalyxptychospermatis*	ZHKUCC 21 0008^T^	MZ493352	OK569894	Phukhamsakda et al. (2022)
*Endocalyxptychospermatis*	ZHKUCC 21 0009^T^	MZ493353	OK569895	Phukhamsakda et al. (2022)
*Endocalyxptychospermatis*	ZHKUCC 21 0010^T^	MZ493354	OK569896	Phukhamsakda et al. (2022)
*Longiappendisporachromolaenae*	MFLUCC 17-1485^T^	MT214370	MT214464	Mapook et al. (2020)
*Requienellafraxini*	RS7	KT949911	NA	Jaklitsch et al. (2016)
*Requienellafraxini*	CBS 140475	NR138415	NA	Jaklitsch et al. (2016)
*Requienellaseminuda*	CBS 140502^T^	NR154630	MH878683	Jaklitsch et al. (2016)
*Seynesiaerumpens*	SMH 1291	NA	AF279410	Bhattacharya et al. (2000)

Notes: Type specimens are marked with T; “NA”: indicates no sequence available in GenBank; newly generated sequences are indicated in bold.

**Table 2.** Taxa and corresponding GenBank accession numbers of sequences used in the phylogenetic analysis of Fig. 2.

Species	Strain number	GenBank Accession Numbers				References
		ITS	LSU	*tub2*	*tef1α*	
*Alloanthostomellarubicola*	MFLUCC 16-0479	KX533455	KX533456	NA	NA	Daranagama et al. (2016)
*Anthostomellaobesa*	MFLUCC 14-0171	KP297405	KP340546	KP406616	NA	Daranagama et al. (2015)
*Melanographiumphoenicis*	MFLUCC 18-1481^T^	MN482677	MN482678	NA	MN481518	Hyde et al. (2020b)
*Melanographiumsmilacis*	MFLU 21-0075	MZ538514	MZ538548	NA	NA	Boonmee et al. (2021)
*Nigropunctatabambusicola*	MFLU 19-2134^T^	MW240662	MW240592	NA	MW759547	Samarakoon et al. (2022)
*Nigropunctatabambusicola*	MFLU 19-2145^T^	MW240664	MW240594	NA	MW759548	Samarakoon et al. (2022)
*Nigropunctatacomplanata*	HHUF 30674^T^	LC760560	LC760580	NA	LC760613	Sugita et al. (2024)
*Nigropunctatacomplanata*	HHUF 30675^T^	LC760561	LC760581	NA	LC760614	Sugita et al. (2024)
*Nigropunctatacomplanata*	HHUF 30676^T^	LC760562	LC760582	NA	LC760615	Sugita et al. (2024)
*Nigropunctatacomplanata*	HHUF 30677^T^	LC760563	LC760583	NA	LC760616	Sugita et al. (2024)
*Nigropunctatahydei*	CMUB 40018^T^	OR507150	OR507163	NA	NA	Samarakoon et al. (2023)
*Nigropunctatahydei*	MFLU 23-0410^T^	OR507151	OR507164	NA	NA	Samarakoon et al. (2023)
*Nigropunctatakhalidii*	GMB1156^T^	PP153389	NA	PP209114	NA	Li et al. (2024)
*Nigropunctatanigrocircularis*	MFLU 19-2130^T^	MW240661	MW240591	MW775612	MW759546	Samarakoon et al. (2022)
*Nigropunctatasaccata*	MFLU 19-2144^T^	MW240663	MW240593	MW775613	NA	Samarakoon et al. (2023)
*Nigropunctatasaccata*	MFLU 18-0804	MW240658	MW240588	MW775611	NA	Samarakoon et al. (2023)
*Nigropunctatathailandica*	MFLU 19-2118^T^	MW240659	MW240589	NA	MW759544	Samarakoon et al. (2022)
*Nigropunctatathailandica*	HKAS 106975	MW240660	MW240590	NA	MW759545	Samarakoon et al. (2022)
***Nigropunctataxiaohensis***	**GMB4503^T^**	**PQ066510**	**PQ066518**	**PQ083530**	**PQ083532**	**The study**
***Nigropunctataxiaohensis***	**GMB4552**	**PQ066511**	**PQ066519**	**PQ083531**	**PQ083533**	**The study**
*Pseudoanthostomellaconorum*	CBS 119333	EU552099	EU552099	NA	NA	Daranagama et al. (2016)
*Pseudoanthostomelladelitescens*	MFLUCC 16-0477	KX533451	KX533452	KX789490	NA	Daranagama et al. (2016)
*Pseudoanthostomellapini-nigrae*	MFLUCC 16-0478^T^	KX533453	KX533454	NA	NA	Daranagama et al. (2016)
*Pseudoanthostomellapini-nigrae*	MFLU 18-0877	MW240654	MW240584	MW820918	MW759541	Daranagama et al. (2016)
*Pseudoanthostomellapini-nigrae*	MFLU 15-3608	MW240655	MW240585	MW820919	MW759542	Daranagama et al. (2016)
*Pseudoanthostomellapini-nigrae*	HKAS 102309	MW240656	MW240586	MW820920	NA	Daranagama et al. (2016)
*Pseudoanthostomellasenecionicola*	MFLUCC 15-0013	MW240674	MW240604	MW820913	MW759554	Daranagama et al. (2016)
*Virgarianigra*	CBS 128006	MH864744	MH876180	NA	NA	Vu et al. (2019)

Notes: Type specimens are marked with T; “NA”: indicates no sequence available in GenBank; newly generated sequences are indicated in bold.

## ﻿Results

### ﻿Phylogeny

Analyses 1: Placements of *Amphibambusa* and *Arecophila*

**Figure 1.**
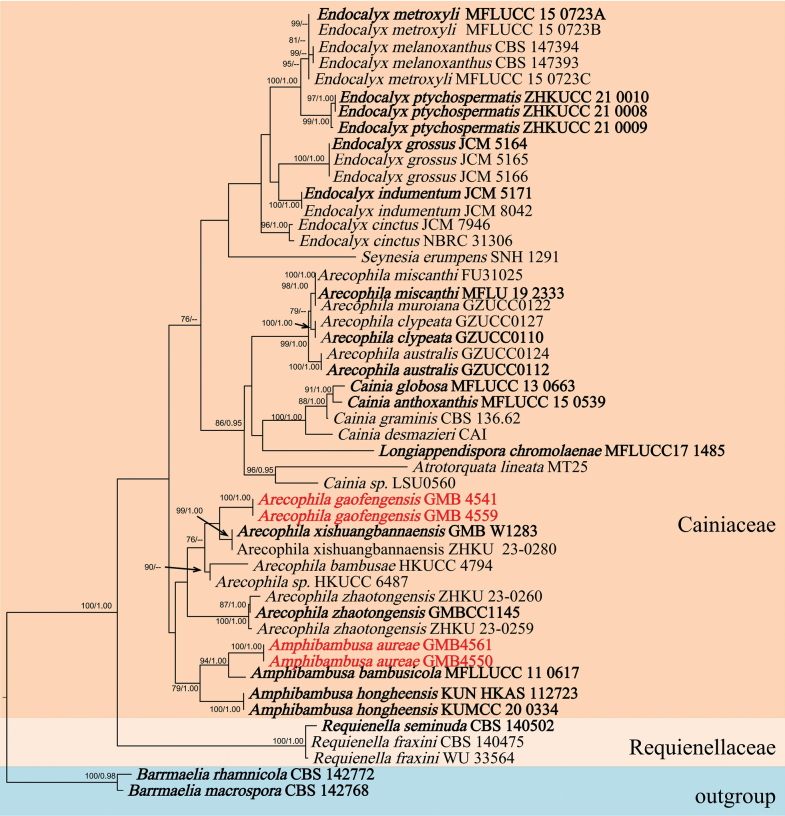
Molecular phylogenetic analysis of *Amphibambusaaureae*, *Arecophilagaofengensis* and related taxa based on a combined ITS and LSU sequences. Bootstrap support values for maximum likelihood (ML) greater than 75% and Bayesian posterior probabilities (BPP) greater than 0.95 are displayed above or below the respective branches (ML/BI). The newly described species are marked red. Holotype and ex-type materials are in bold.

The aligned data set of phylogram (Fig. 1) comprised 1250 (ITS/LSU) characters, after the exclusion of ambiguously aligned regions and long gaps. *Barrmaeliamacrospora* (Nitschke) Rappaz and *B.rhamnicola* Rappaz were chosen as the outgroup taxa. The sequences of our collection *Amphibambusaaureae* formed a clade, exhibiting a firmly established sister relationship with *Amphibambusabambusicola* D.Q. Dai & K.D. Hyde (94/1.00 ML/BI, Fig. 1). Newly generated sequences from *Arecophilagaofengensis* strains formed a sister branch to those of *A.xishuangbannaensis* L.S. Han & D.Q. Dai with a low support value (69/0.86 ML/BI, Fig. 1). *Amphibambusaaureae* and *Arecophilagaofengensis* are described as two new species.

#### ﻿Analyses 2: Placement of *Nigropunctata*

**Figure 2.**
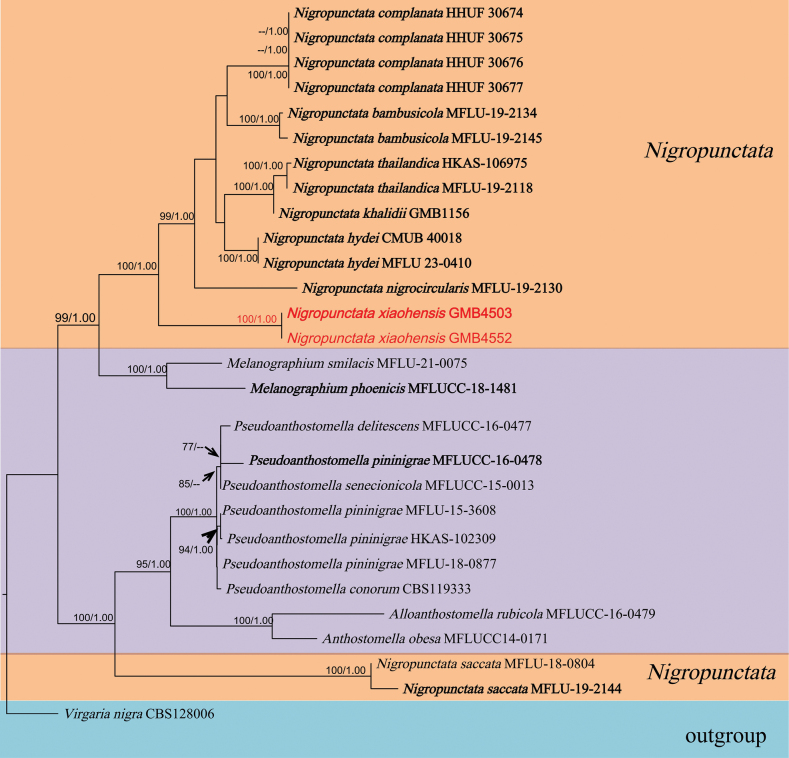
Molecular phylogenetic analysis of *Nigropunctata* and related taxa based on a combined ITS, LSU, *tub2* and *tef1α* sequences. Bootstrap support values for maximum likelihood (ML) greater than 75% and Bayesian posterior probabilities (BPP) greater than 0.95 are displayed above or below the respective branches (ML/BI). The newly described species are marked red. Holotype and ex-type materials are in bold.

The aligned dataset of *Nigropunctata* (Fig. 2) comprised 2730 (ITS/LSU/*tub2/tef*1α) characters, after exclusion of ambiguously aligned regions and long gaps. *Virgarianigra* (Link) Nees was chosen as the outgroup taxon. In the phylogram (Fig. 2), the sequences of our collection *Nigropunctataxiaohensis* formed a well-supported (100/1.00, ML/BI) distinct clade on the basal of *Nigropunctata. Nigropunctataxiaohensis* is described as a new species.

### ﻿Taxonomy

#### ﻿ Amphibambusa

Taxon classificationFungiAmphisphaerialesAmphisphaeriaceae

D.Q. Dai & K.D. Hyde Fungal Diversity 72: 9, 2015.

AB8F6706-7232-54DE-AB1C-E62F00C1C914

550940

##### Notes.

The genus *Amphibambusa* was introduced by Liu et al. (2015) which is characterized by immersed, solitary, scattered, globose to subglobose ascomata, ostiole at the centre, surrounded by white margin, unitunicate, cylindrical, short-pedicellate asci with a J+, subapical ring, and fusiform, subhyaline, longitudinally striated, 1-septate ascospores surrounded by a gelatinous sheath. Currently, the genus comprises two species: *A.hongheensis* H.B. Jiang & Phookamsak and *A.bambusicola* D.Q. Dai & K.D. Hyde (Liu et al. 2015, Jiang et al. 2021a). In this study, we introduce a new species of *Amphibambusa* from Guangxi Zhuang Autonomous Region, China.

#### ﻿ Amphibambusa aureae

Taxon classificationFungiAmphisphaerialesAmphisphaeriaceae

X. Zhou, K. Habib & Q. R. Li sp. nov.

7AC9BBE6-503D-5687-88F2-B2DF47BB1213

853721

Fig. 3

##### Etymology.

Named after the host-specific epithet “*Phyllostachysaureae* Rivière & C. Rivière” from which the fungus was isolated.

##### Type.

China • Guangxi Zhuang Autonomous Region, Liangfengjiang Forest Park (22°43'24.91"N, 108°26'56.39"E), altitude: 99 m, on *Phyllostachysaureae*, 15 August 2023, Xin Zhou, Wenyu Zeng, 2023LFJ9 (GMB4550, holotype; GMBC4550, ex-type); *ibid*KUN-HKAS 134919, isotype.

##### Description.

Saprobic on dead culms of bamboo, forming black circular spots on the host surface. ***Sexual morph***: Ascomata 660–860 μm wide, 520–630 μm high, immersed under host epidermis, solitary, scattered, globose to subglobose, visible as a black dot, ostiole at the center, with a neck, with an underdeveloped clypeus. Ostioles are centrally located, black, surrounded by white margin. Peridium 13–30 μm thick, outer brown to hyaline inner, cells ***textura angularis***. Paraphyses 2–4.8 μm (x̄ = 3.7 μm, n = 20) wide, longer than the asci, numerous, filamentous, colorless, branched, septate. Asci 90–190 × 9–18 μm (x̄ = 148.5 × 13.1 μm, n = 20), 8-spored, unitunicate, cylindrical, short-pedicellate, apically rounded, with a J+ subapical ring, 1.4–1.9 × 2.5–3.6 μm (x̄ = 1.7 × 3.1 μm, n = 6). Ascospores 15–22.5 × 5–7.9 μm (x̄ = 19 × 6.6 μm, n = 40), L/W 3.4, 1–2 seriate, fusiform, subhyaline, 1-septate in the middle, slight constricted at the septum, with round ends, with longitudinal striations along the entire length of the ascospore, and enveloped by a gelatinous sheath 2.5–7 μm (x̄ = 5.2 μm, n = 20), lacking appendage. ***Asexual morph***: Undetermined.

##### Culture characteristics.

Cultured on PDA medium at 27 °C for 4–5 weeks, the colony diameter measures 4–4.5 cm, round, slightly raised in the center, with a neat margin. The mycelium at the colony edge is degraded, appearing white and glossy. A portion of the colony center is brown.

##### Paratype.

CHINA • Guangxi Zhuang Autonomous Region, Liangfengjiang Forest Park (22°43'20.90"N, 108°26'33.52"E), altitude: 99 m, on *Phyllostachysaureae*, 15 August 2023, Xin Zhou, Wenyu Zeng, 2023LFJ190 (GMB4561; paratype; GMBC4561, ex-paratype).

##### Notes.

In the phylogram, *Amphibambusaaureae* (ex-type: GMBC4550) clustered in a distinct clade close to *A.bambusicola* D.Q. Dai & K.D. Hyde (ex-type: MFLLUCC 11–0617). The genus *Amphibambusa* is represented by two species, *A.hongheensis* H.B. Jiang & Phookamsak and *A.bambusicola*. *Amphibambusaaureae* shares similarities with both species, such as ascomata immersed in a black clypeus, ostiolar openings surrounded by a white margin, cylindrical asci with a J+ subapical ring, and fusiform, longitudinally striated ascospores enveloped by a distinct mucilaginous sheath (Liu et al. 2015, Jiang et al. 2021a). However, *A.aureae* can be distinguished from *A.bambusicola* by its smaller ascospores (15–22.5 × 5–7.9 μm compared to 25–27 × 5.5–6 μm in *A.bambusicola*) (Liu et al. 2015). Additionally, ascospores of *A.aureae* have rounded ends and are slightly constricted at the septum, whereas those of *A.bambusicola* have pointed end cells and are deeply constricted at the septum. *Amphibambusahongheensis* differs from *A.aureae* by having smaller asci (118–160 × 14–18 μm vs. 90–190 × 9–18 μm) and larger ascospores (25.5–33 × 5.5–7.2 μm vs. 15–22.5 × 5–7.9 μm) (Jiang et al. 2021a).

**Figure 3.**
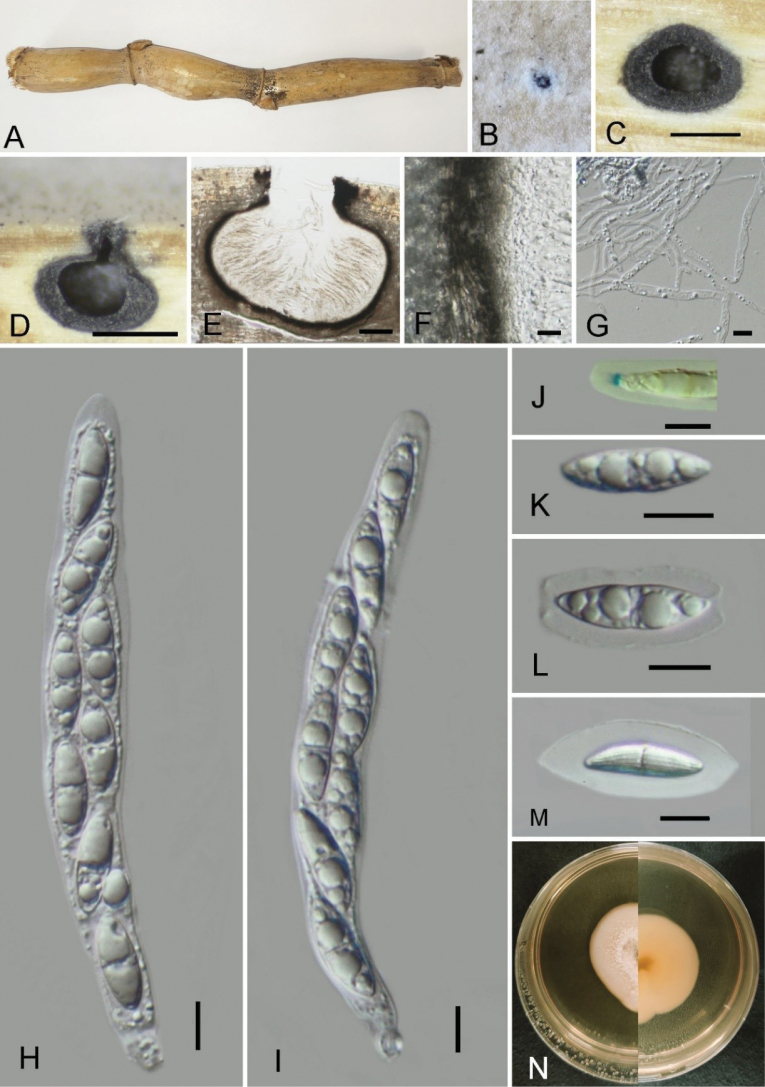
*Amphibambusaaureae* (GMB4550, holotype) **A** type material **B** ascoma immersed under the surface of host **C** cross-section of ascoma **D, E** longitudinal sections of ascomata **F** peridium **G** paraphyses **H, I** asci **J** a J+ subapical ring bluing in Melzer’s reagent **K–M** ascospores **N** culture on PDA. Scale bars: 0.5 mm (**C, D**); 100 μm (**E**); 10 μm (**F–M**).

### ﻿Key to the *Amphibambusa* species

**Table:** 

1	Ascospore > 22 µm long	**3**
2	14.7–21.47 μm long ascospore	***A.aureae***
3	Ascospore 25–27 μm long, with pointed end cells, deeply constricted at the septum	***A.bambusicola***
–	Ascospore 25.5–33 μm long, with round end cells, and slightly constricted at the septum	***A.hongheensis***

#### ﻿ Arecophila

Taxon classificationFungiXylarialesCainiaceae

K.D. Hyde, Nova Hedwigia 63(1–2): 82 (1996)

481E7D78-C19E-528F-B25E-55BD97BAD6C8

27653

##### Notes.

The genus *Arecophila* was introduced by Hyde (1996). The genus is characterized by immersed ascomata with blackened clypeus, ostiole at the centre, unitunicate, long-cylindrical asci with a J+, apical ring, and 1-septate ascospores with striations, and covered with a thick mucilaginous sheath (Hyde 1996; Li et al. 2022; Han et.2024). In this study, we introduce a new species of *Arecophila* from Guizhou Province, China.

#### ﻿ Arecophila gaofengensis

Taxon classificationFungiXylarialesCainiaceae

X. Zhou, K. Habib & Q. R. Li sp. nov.

6557735A-E282-5AF8-9A5F-D448B5854DD3

853722

Fig. 4

##### Etymology.

The specific epithet “*gaofengensis*” refers to the geographical location, Gaofeng Village, where the holotype specimen was collected.

##### Type.

China • Guizhou Province, Anshun City, Pingba District, Gaofeng Town, 26°33'96.54"N, 106°54'20.37"E, altitude: 1250 m, on dead culms of bamboo, 30 October 2023, Yulin Ren, 2023GFZ15 (GMB4541, holotype; GMBC4541, ex-type); *ibid*KUN-HKAS 134920, isotype.

##### Description.

Saprobic on the surface of dead bamboo culms, forming black round spots. ***Sexual morph***: Ascomata 400–600 µm high, 600–900 µm diam, globose to subglobose, solitary, scattered, sometimes gregarious, immersed beneath blackened clypeus; clypeus well developed, black, coriaceous, ostiole at the center, weakly papillate. Peridium 13–20 μm wide, composed of thick walled, hyaline to brown cells, ***texture angularis***. Paraphyses 2–3 µm (x̄ = 2.6 µm, n = 20) wide, hyaline, numerous, filamentous, branched, septate. Asci 126–210 × 10–13.5 µm (x̄ = 165 × 12.5 µm, n = 20), 8-spored, unitunicate, long-cylindrical, short-pedicellate, with a J+, trapezoidal shape apical ring, bluing in Melzer’s reagent, 2.2–3.4 μm high, 3.6–4.2 μm diam. Ascospores 19–24.5 × 7–9.5 µm (x̄ = 21.6 × 7.8 µm, n = 30), uniseriate, fusiform, brown, 1-septate, septate at the center, slightly constricted septum, tapering at the ends, with longitudinal and sulcate striations, covered with a thick mucilaginous sheath measuring 3–8 µm (x̄ = 6.3 µm, n = 10). ***Asexual morph***: Undetermined.

##### Culture characteristics.

Ascospores germinating on PDA within 36 hours and germ tubes produced from upper cells. Colonies growing fast on PDA, reaching 2 cm in 1 week at 28 °C, effuse, velvety to hairy, nearly circular, irregular at the margin, white from above, pale yellowish white from below. Mycelium immersed in the media, composed of branched, septate, smooth-walled, hyaline, hyphae.

**Figure 4.**
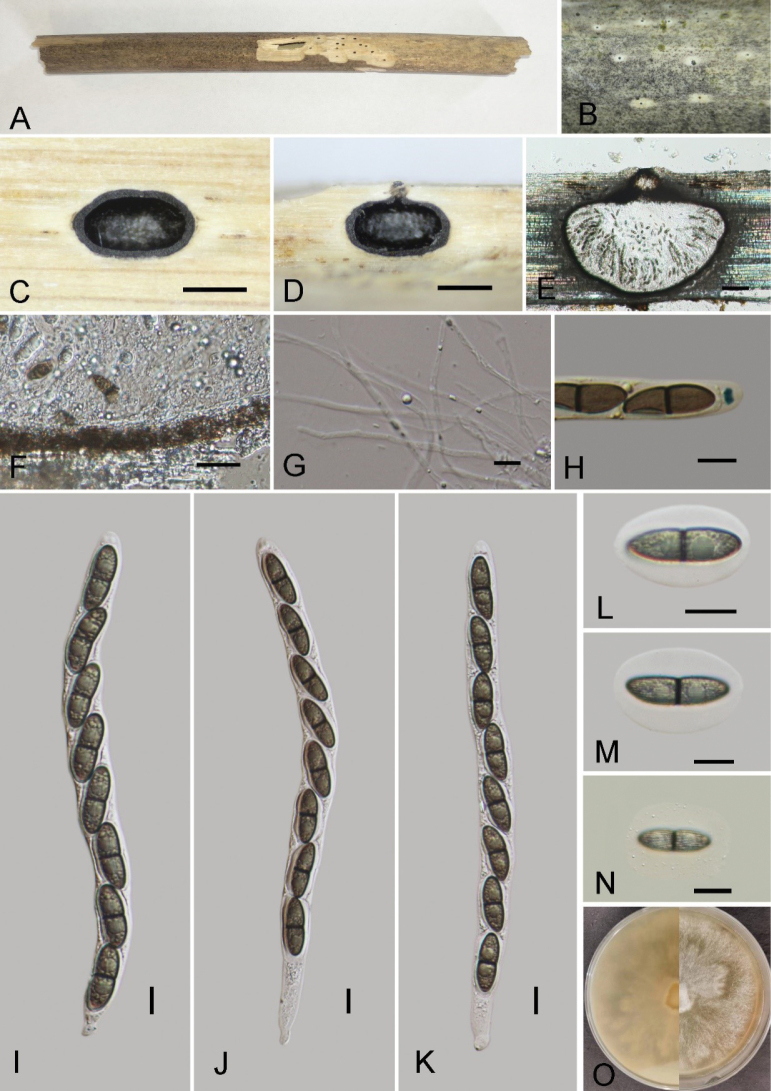
*Arecophilagaofengensis* (GMB4541, holotype) **A, B** ascomata immersed in bamboo host **C** Cross-section of ascoma **D, E** longitudinal sections of ascomata **F** peridium **G** paraphyses **H** a J+ subapical ring staining by Melzer’s reagent **I**–**K** asci with ascospores **L–N** ascospores surrounded by a gelatinous sheath. Scale bars: 0.5 mm (**B–D**); 100 μm (**E**); 10 μm (**F–N**).

##### Paratype.

China • Guizhou Province, Anshun City, Pingba District, Gaofeng Town, 26°33'95.44"N, 106°54'30.27"E, altitude: 1250 m, on dead culms of bamboo, 30 October 2023, Yulin Ren, 2023GFZ530 (GMB 4559; paratype; GMBC4559, ex-paratype).

##### Notes.

In the phylogram (Fig. 1), *Arecophilagaofengensis* formed a sister branch with *A.xishuangbannaensis* L.S. Han & D.Q. Dai with a low bootstrap values (69/0.86 ML/BI, Fig. 1). *Arecophilagaofengensis* differs from *A.xishuangbannaensis* by its smaller ascospores (19–24.5 × 7–9.5 µm vs. 23–27 × 8.5–9.5 μm) and smaller asci (126–210 × 10–13.5 µm vs. 180–270 × 12–14 μm) (Han et al. 2024). The analysis of ITS sequences for these two species reveals a sequence length of 471 base pairs, with a 92.8% similarity, and a 2.1% gap presence, indicating 437 matching positions. Morphologically, the new taxon is close to *A.bambusae*, but can be distinguished from *A.bambusae* by having larger asci (126–210 × 10.3–13.7 µm vs. 132.5–140 × 7.5–8 µm) and wider ascospores (19–24.5 × 7.1–9.5 µm vs. 19–22.5 × 5.5–7 µm) (Umali et al. 1999). Morphologically, the new species also resembles *A.muroiana* (I. Hino & Katum.) You Z. Wang et al. However, clypeus is absent in *A.muroiana* (Li et al. 2022), while blackened clypeus was observed in *A.gaofengensis*. So, here we introduced it as a new species of *Arecophila*.

#### ﻿ Nigropunctata

Taxon classificationFungiXylarialesXylariaceae

Samarak. & K.D. Hyde, Fungal Diversity 112: 68, 2022.

6F51C819-D3F5-5A31-A286-06DDFC581E13

558737

##### Notes.

The genus *Nigropunctata*, typified by *N.bambusicola* Samarak. & K.D. Hyde, has recently been classified into Xylariales. The genus is characterized by immersed, solitary or scattered ascomata appearing as small black dots, unitunicate, cylindrical asci with a J+, discoid apical ring (Samarakoon et al. 2022). The genus is represented by seven species (https://www.indexfungorum.org/Names/Names.asp; Accessed June 21, 2024). In this study, we introduce a new species of *Nigropunctata* from China.

#### ﻿ Nigropunctata xiaohensis

Taxon classificationFungiXylarialesXylariaceae

X. Zhou, K. Habib & Q. R. Li sp. nov.

55B26C59-0A3C-593F-8F39-A210811FA55A

853723

Fig. 5

##### Etymology.

The specific epithet “xiaohensis” refers to the geographical location, Xiaohe Village, where the holotype specimen was collected.

##### Type.

China • Guizhou Province, Guiyang City, Huaxi District, Xiaohe Village, (25°33'10.46"N, 105°38'22.57"E), altitude: 120 m, on bamboo, 1 June 2023, Xin Zhou, Wenyu Zeng, 2023XHC1 (GMB4503, holotype, no culture was obtained); *ibid*KUN-HKAS 134921, isotype.

##### Description.

Saprobic on decaying bamboo culms. ***Sexual morph***: Ascomata 320–380 × 340–400 μm (x̄ = 352.7 × 360 μm, n = 10), immersed, solitary or scattered, appearing as small black dots, solitary, in cross-section globose to subglobose with a flattened base. Ostioles centrally, slightly, papillate, black, flush with the surface of the host. Peridium 15–25 µm thick, comprised of several layers, composed of thick-walled, dense, brown to hyaline, cells of ***textura angularis***. Paraphyses 2.8–4.3 μm (x̄ = 3.6 μm, n = 20) wide, longer than the asci, numerous, filamentous, curving, contain white intracellular material. Asci 85.5–140 × 11–18.5 μm (x̄ = 120.2 × 15.5 μm, n = 20) 8-spored, unitunicate, cylindrical, short-pedicellate, apically rounded, with a J+, discoid apical ring, measures 1.3–2.4 μm high, 3.5–5.0 μm wide (x̄ = 1.8 × 4.4 μm, n = 10). Ascospores 11–21 × 6.5–10.5 μm (x̄ = 17.8 × 8.1 μm, n = 30), L/W 2.2, uniseriate, unicellular, ellipsoid to broadly ellipsoid, dark brown to black, with rounded ends, covered with a thick mucilaginous sheath measuring 5–8 µm (x̄ = 6.2 µm, n = 10), with a germ slit extending across the entire spore. ***Asexual morph***: Undetermined.

**Figure 5.**
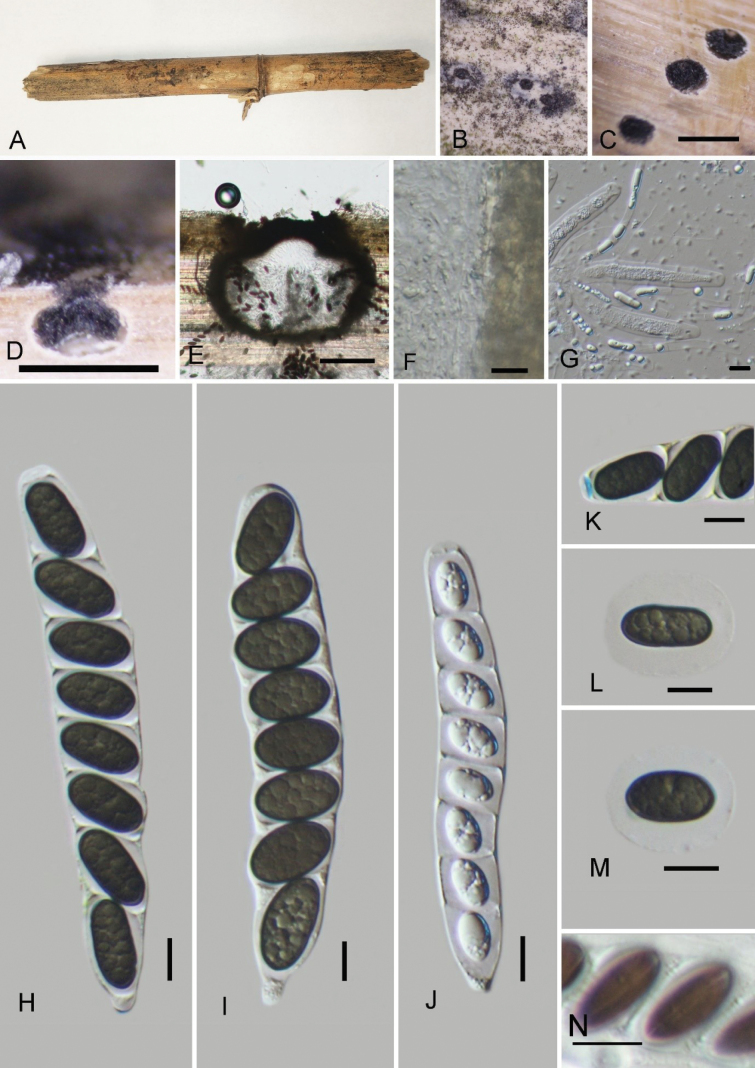
*Nigropunctataxiaohensis* (GMB4503, holotype) **A** material **B** ascoma on the surface of host **C** cross-section of ascoma **D, E** longitudinal sections of ascomata **F** peridium **G** paraphyses **H**–**J** asci **K** a wedge-shaped, J+ apical ring bluing in Melzer’s reagent **L, M** ascospores **N** ascospores with germ slits. Scale bars: 0.5 mm (**C, D**); 100 μm (**E**); 10 μm (**F**–**N**).

##### Paratype.

China • Guizhou Province, Guiyang City Huaxi District, Xiaohe Village (25°33'20.34"N, 105°38'32.23"E), altitude: 120 m, on bamboo, 4 June 2023, Xin Zhou, Wenyu Zeng, 2023XHC340 (GMB4552, paratype).

##### Notes.

In the phylogram (Fig. 2), *Nigropunctataxiaohensis* formed a separate clade in *Nigropunctatas. str.* Morphologically, *N.xiaohensis* resembles *N.complanate* R. Sugita & Kaz. Tanaka (Sugita et al. 2024) as both share similar size ascospore. However, *N.complanate* is distinguished by thick clypeus (75–90 μm high, 270–410 μm diam.), larger asci (130–175 × 13–20 μm), and an inverted hat-shaped apical ring. The ITS sequences analysis of *N.complanate* and *N.xiaohensis* reveals a sequence length of 496 base pairs, with an 84.3% identity, and 9.1% gap presence. *Nigropunctatanigrocircularis* Samarak. & K.D. Hyde differs in having larger ascomata (450–535 × 455–560 μm), longer asci (125–170 μm) and smaller ascospore averaging 15.5 × 6.4 μm with a 3–4.5 μm mucilaginous sheath (Samarakoon et al. 2022). The type species of the genus, *N.bambusicola* Samarak. & K.D. Hyde differs in having smaller ascomata measuring 285–315 × 260–340 μm, smaller discoid-inverted hat-shaped ascal apical rings (1.7–2 × 4–4.8 μm), and ascospores measuring 13.5–17 × 5.5–9.5 μm, with a 2–6 μm mucilaginous sheath (Samarakoon et al. 2022). A recently reported new species from China, *N.khalidii* Y. P. Wu & Q. R. Li, differs by possessing larger ascomata (608–782 × 762–830 μm vs. 320–380 × 340–400 μm in *N.xiaohensis*), larger asci (146–173 × 8.6–13.6 µm vs. 85.5–140 × 11–18.5 μm in *N.xiaohensis*), and slightly smaller ascospores (14.8–18 × 6.3–9 µm) lacking a germ slit (Li et al. 2024).

### ﻿Key to the *Nigropunctata* species

**Table:** 

1	Ascospores lacking a germ slit	**4**
2	Ascospores with germ slit	**5**
3	Lacking mucilaginous sheath around ascospores	***N.saccata***
4a	peridium 11–16 µm wide, ascomata 606–782 × 762–830 µm	***N.khalidii***
4b	Peridium 16.5–31 µm wide, ascomata 400–520 × 485–575 µm	***N.hydei***
5a	Ascomata > 450 µm diam	**6**
5b	Ascomata 260–340 μm diam, asci 95–140 µm long, ascal apical apparatus 1.7–2 × 4–4.8 µm	***N.bambusicola***
5c	Ascomata 390–450 µm diam, asci 130–175 µm long, ascal apical apparatus 2.5–3 × 4.5–5 µm	***N.complanata***
5d	Ascomata 340–400 µm diam, asci 85.5–140 µm long, ascal apical apparatus 1.3–2.4 × 3.5–5 µm	***N.xiaohensis***
6a	Ascomata 450–535 × 455–560 μm, ascal apical apparatus 3.2–3.6 µm wide	***N.nigrocircularis***
6b	Ascomata 615–830 × 770–965 μm, ascal apical apparatus 4.5–6 μm wide	***N.thailandica***

## ﻿Discussion

In this paper, three new species of *Amphibambusa*, *Arecophila*, and *Nigropunctata* associated with bamboo were introduced, which were collected from karst areas of China. Recent studies have expanded our understanding of bambusicolous fungi from southern China. Han et al. (2024) introduced three new species from the family Cainiaceae, including a novel genus *Paramphibambusa* and two new *Arecophila* species. Jiang et al. (2021b) described two new species, *Occultibambusahongheensis* and *Seriascomabambusae*, and reported *Occultibambusakunmingensis* from new habitats. Yu et al. (2023) identified three new species in the Savoryellaceae family and two new records from Sichuan Province. Our discoveries have enriched the research on bambusicolous fungal diversity in southern China.

*Amphibambusa* has a widespread distribution, reported in both Thailand and China. All known species of *Amphibambusa* have been found exclusively on decorating bamboo, indicating a potential host specificity (Liu et al. 2015; Jiang et al. 2021a). Phylogenetic analysis conducted in this study reveals a close relationship between *Amphibambusa* and *Arecophila*. However, *Amphibambusa* possesses hyaline ascospores pointed at both ends, which distinguishes it from *Arecophila* (Liu et al. 2015). The longitudinal stripes on the surface of *Amphibambusa* ascopores are not easily visible under an optical microscope and can be easily overlooked. Special attention should be paid when observing and describing morphology. Here one new species of *Amphibambusaaureae* was introduced as the third species of the genus.

Currently, there are 20 *Arecophila* epithets in Index Fungorum (http://www.indexfungorum.org/Names/Names.asp, July 2024), but only six species and one strain of *Arecophila* sp. have molecular data on Genbank. *Arecophila* clustered into two clades through phylogenetic analysis (Li et al. 2022, Han et al. 2024). Our study also identifies *Arecophila* as comprising two clades. Morphologically, we cannot find a clear difference between these two clades. At the same time, the morphological characteristics of the species in both branches conform to definitions of *Arecophila* (Hyde 1996). This may indicate that *Arecophila* is a polyphyletic group that has undergone convergent evolution. It may also indicate that the genes currently used to construct phylogenetic trees cannot serve as good DNA barcoding for distinguishing *Arecophila* from its approximate genera. In summary, the use of more samples, gene sequences, and morphological features is essential for the future accurate identification of *Arecophila*.

Ascospores are the main identifying feature of ascomycetous fungi (Webster and Weber 2007). Currently, there are eight *Nigropunctata* species published including our new introduction. However, the shapes, dimensions, and colors of the ascospores of all species in the genus *Nigropunctata* are similar with very little variation (Samarakoon et al. 2022, 2023; Li et al. 2024; Sugita et al. 2024). The presence or absence of germ slits and mucilaginous sheaths of ascospores is used as the main basis for distinguishing *Nigropuntatakhalidii*, *N.hydei*, *N.saccata* from similar species (Samarakoon et al. 2023; Li et al. 2024). In terms of ascospores’ size, the mean value of ascospores of all eight species was 15–18 μm. For example, the ascospores of *N.thailandica* measure 15–18.5 × 7–11.5 μm (mean = 17 × 9 μm, n = 25), while those of *N.complanata* measure 14.5–19.5 × 7.5–10 μm (Samarakoon et al. 2022; Sugita et al. 2024). The averages of the ascospore sizes of these two species differed by only 0.1 µm. The ascospore colors of all eight species are brown to dark brown, and the ascospore shapes of all eight species are ellipsoidal (Samarakoon et al. 2022; Samarakoon et al. 2023; Li et al. 2024; Sugita et al. 2024). Except for the ascospores, there are also relatively small morphological differences among the *Nigropunctata* species (Samarakoon et al. 2022; Samarakoon et al. 2023; Li et al. 2024; Sugita et al. 2024). However, there are significant differences in their DNA sequences. Hence, we believe that DNA sequence should be a primary feature for the species identification of *Nigropunctata*.

## Supplementary Material

XML Treatment for Amphibambusa

XML Treatment for Amphibambusa aureae

XML Treatment for Arecophila

XML Treatment for Arecophila gaofengensis

XML Treatment for Nigropunctata

XML Treatment for Nigropunctata xiaohensis

## ﻿Additional information

### Author ORCIDs

Kamran Habib https://orcid.org/0000-0003-2572-0306

Yulin Ren https://orcid.org/0009-0003-9063-425X

Jichuan Kang https://orcid.org/0000-0002-6294-5793

Qirui Li https://orcid.org/0000-0001-8735-2890

### Data availability

The datasets generated during and/or analyzed during the current study are available in the MycoBank repository (included in the manuscript), and GenBank (included in Tables 1, 2). And also, the datasets generated during and/or analyzed during the current study are available from the corresponding author on reasonable request.
